# Short-term antibiotic therapy for the most common bacterial respiratory infections in infants and children

**DOI:** 10.3389/fphar.2023.1174146

**Published:** 2023-06-06

**Authors:** Nicola Principi, Giovanni Autore, Alberto Argentiero, Susanna Esposito

**Affiliations:** ^1^ Università Degli Studi di Milano, Milan, Italy; ^2^ Pediatric Clinic, Pietro Barilla Children’s Hospital, Department of Medicine and Surgery, University of Parma, Parma, Italy

**Keywords:** acute otitis media, acute streptococcal pharyngitis, antibiotics, community-acquired pneumonia, short-term therapy

## Abstract

Overuse and misuse of antibiotics have strongly accelerated the progressive increase in bacterial antimicrobial resistance (AMR). The evidence that antimicrobial selective pressure was greater the longer the antibiotic therapy was continued has led some experts to reconsider duration of antibiotic therapy testing the use of short-term drug administration. If as effective as long-term therapy, short-term therapy could have been an easy measure to limit AMR emergence. In the present narrative review, whether present knowledge on short-term therapy of acute streptococcal pharyngitis (ASF), acute otitis media (AOM) and mild to moderate community-acquired pneumonia (CAP) allows systematic use of short-term therapy in infants and children with these diseases is discussed. Literature analysis showed that reducing the duration of antibiotic therapy for some of the most common pediatric respiratory infections could be a valid measure to contain the antibiotic abuse and the consequent impact on the emergence of AMR. Several data seem to indicate that this type of intervention is possible, as short-term therapy has been found as effective as the traditionally recommended long-term therapy in some cases of ASF, AOM and mild to moderate CAP. However, further studies are needed to better characterize infants and children who can have benefit with short-term antibiotic therapy in common bacterial respiratory infections.

## 1 Introduction

The progressive increase in bacterial antimicrobial resistance (AMR) has made it difficult to treat several infectious diseases that were once easily cured with the most widely used antibiotics (Fanelli et al., 2020). Bacterial infections have again become a severe medical problem because more frequently associated to longer hospital stays, higher medical costs and increased risk of death or development of persistent disability (Aljeldah, 2022). In 2019, a comprehensive analysis of the burden of AMR, covering estimates for 204 geographic areas, has shown that AMR was associated with 4.95 million deaths. (Antimicrobial Resistance Collaborators, 2022). Of these, 1.27 million deaths were directly attributable to AMR, thus making AMR the third most important cause of death after ischemic heart disease and stroke that year (GBD, 2019 Diseases and Injuries Collaborators, 2020). Moreover, it was shown that AMR had caused the loss of 47,600 years of life and that survivors had to live with disabilities for a total of 275 years. Children, particularly neonates and infants, pay a very high price for AMR. WHO estimated that every year in the world about 30% of deaths due to multi-resisting bacteria (MRB) occur in newborns (Romandini et al., 2021). Similar findings were reported in Europe (Cassini et al., 2019) when the prevalence of death secondary to AMR in children aged less than 1 year was calculated.

Emergence of bacterial AMR occurs naturally, usually through genetic changes. However, overuse and misuse of antibiotics have strongly accelerated this process and remain the most critical factors for emergence of AMR (English and Gaur, 2010; Akram et al., 2023). Improving antibiotic use through high quality surveillance and usage guidelines has been considered the most important solution to face AMR problems. Several national governments and scientific institutions, such as the European Union Commission (European Commission, 2023), the United States Government (ASPE. National, 2023) and the World Health Organization (World Health Organization, 2023a), have initiated or accelerated the development of action plans to combat AMR. Official guidelines include specific criteria for antibiotic prescribing practices to ensure a rational use of antibiotics and reduce AMR.The antibiotic recommendations, within the guidelines, include antibiotic selection, doses, routes of administration and duration for each individual infectious disease. However, whereas suggestions for the other variables were generally based on controlled investigations, those concerning duration of antibiotic therapy were often based on historical habits without scientific evidence. Antibiotic courses of no less than 7–10 days were generally recommended. This duration was considered sufficient to eradicate infectious pathogen and preventing emergence of AMR (Rice, 2008). In recent years more evidence has emerged associating prolonged duration of therapy with greater antimicrobial selective pressure. This evidence has led some experts to reconsider and reducing the duration of antibiotic therapy (Costelloe et al., 2010; Teshome et al., 2019; Curran et al., 2022). Studying short-course therapy is a simple intervention to study, thus limiting AMR emergence if proven equally efficacious to long-course. In fact, several studies have already been completed and paved the way for the new standards of care used in clinical practice today (Smith et al., 2020; Davar et al., 2022; Lee et al., 2023).

Children are among the subjects more frequently treated with antibiotics. The overuse of antibiotics in children is considered one of the highest contributors to AMR development (De Luca et al., 2016). The implementation of antibiotic stewardship programs in pediatrics are essential (Principi and Esposito, 2016; Zay Ya et al., 2023). Despite mainly of viral origin, respiratory infections due to bacteria are common among children (Papan et al., 2022). Use of short-term therapy in some of these conditions could improve antibiotic use in children, simultaneously reducing AMR, drug-related adverse events, and economic cost of therapy (Esposito et al., 2011). We review the present knowledge on short-term therapy of acute streptococcal pharyngitis (ASF), acute otitis media (AOM) and mild to moderate community-acquired pneumonia (CAP).

## 2 Acute streptococcal pharyngitis (ASF)

Acute pharyngitis due to *Streptococcus pyogenes* (*Sp*) is frequently diagnosed. The incidence rate has been estimated to be 22.1 episode per 100 child-years, accounting for more than a quarter of all the pediatric sore throat episodes (Miller et al., 2022). Despite many cases can spontaneously solve, official guidelines worldwide recommend antibiotic therapy in all of the documented cases of ASF. Antibiotic therapy reduces the spread of the pathogen and the risk of developing acute suppurative and long-term non‐suppurative complications, including acute rheumatic fever (ARF) (Centers for Disease Control and Prevention, 2023). Studies have shown that, compared to placebo, antibiotic prescription to patients with sore throat was associated with shorter duration of throat soreness and fever and a more than two-thirds reduction of the risk of development of ARF (relative risk [RR], 0.22; 95% confidence interval [CI], 0.02–2.08) and other potential complications such as AOM (RR, 0.30; 95% CI, 0.15–0.58), acute rhinosinusitis (RR, 0.48; 95% CI, 0.08–2.76), and peritonsillar abscess (RR, 0.15; 95% CI, 0.05–0.47) (Del Mar et al., 2006).

Official guidelines and experts worldwide agree in defining oral penicillin V or amoxicillin (where penicillin V is lacking) as the drugs of choice to treat children with ASF (Chiappini et al., 2012; Pelucchi et al., 2012; Shulman et al., 2012). These agents, despite largely prescribed for several decades, remain highly effective against *S. pyogenes*, and are safe, well tolerated, cheap and, due to their narrow spectrum of activity, at low risk of favoring development of AMR. Alternatives for children with penicillin allergy are second or third generation oral cephalosporin macrolides or clindamycin (Chiappini et al., 2012; Pelucchi et al., 2012; Shulman et al., 2012). Less agreement there is on the duration of the therapy. In most of the official guidelines a 10-day course of one of the first-line drugs is recommended (Chiappini et al., 2011). However, several experts believe that alternative strategies may need to be employed in order to achieve safe reduction in antimicrobial therapy. These include administering different antibiotics such as oral cephalosporins or macrolides, increasing penicillin V or amoxicillin dosage, modifying their time schedule of administration. The approval by Food and Drug Administration (FDA) of the United States of cefdinir and cefpodoxime for a 5-day course of therapy for ASF is one of the best examples in this regard (Holm et al., 2020).

In the attempt to identify whether duration of therapy for ASF could be shortened without reduction in clinical and microbiological efficacy and increase in drug-related adverse events and disease management costs, an enormous number of studies have been carried out in the last 30 years. Unfortunately, despite some studies have suggested that using some drugs and/or some antibiotic schedules of administration a significant reduction of the duration of antibiotic therapy for ASF could be obtained, a global evaluation of the available data seems to lead to less optimistic conclusions. Several aspects of short-term therapy for ASF are not precisely defined. It is not definitively specified which class of antibiotics, which drug or which dosage or schedule of administration assures the optimal short-term therapy. Moreover, poor attention seems to be paid to the problem of the incidence of adverse events and economic costs of therapies different from those based on penicillin V or amoxicillin for 10 days. All these limitations are clearly evidenced in some systematic reviews and meta-analyses that have been published over time. The meta-analysis of studies carried out until November 2007 included 20 randomized controlled trials (RCTs) enrolling 13,102 patients, mainly children, with ASF documented by a positive rapid *S. pyogenes* test of throat swab (Altamimi et al., 2009). The efficacy of a short-course (3–6 days) of any type of antibiotic was compared with the standard 10-day course of penicillin V. Results seemed to indicate overall favorable effects of short-term therapy. This was found as effective as standard therapy in time of resolution of clinical signs and symptoms of sore throat (odds ratio [OR], 0.80; 95% CI, 0.67–0.94), risk of early clinical (OR, 0.95; 95% CI, 0.83–1.08) and microbiological (OR, 1.08; 95% CI, 0.97–1.20) failure, incidence of late clinical and bacteriological recurrences and development of complications. Only 3 days of low dose azithromycin therapy was less effective than penicillin V and other antibiotics regarding *S. pyogenes* eradication. In addition, short-term therapy was associated with a superior compliance (OR, 0.21; 95% CI, 0.16–0.29), even if with a greater incidence of mild adverse events (OR, 1.85; 95% CI, 1.55–2.21) and a greater (although not exactly quantified) economic cost. Despite these findings, conclusions of the authors were very cautious as the meta-analysis had several severe limitations (Altamimi et al., 2009). Most of the included studies had important methodological flaws leading to relevant enrollment bias. The group of antibiotics prescribed for a short-term period was significantly heterogeneous. Penicillin V was compared to several oral cephalosporins and macrolides given with different administration schedules, making it virtually impossible to establish which antibiotic and which regimen was equally effective or better than penicillin V. Only in 3 out of the 20 studies included in the meta-analysis, duration of follow-up of patients after treatment was long enough to allow evaluation of long-term complication incidence of *S. pyogenes* infection, including ARF. Finally, despite short-term therapy was superior with regard to compliance, it was significantly more expensive.

Further data were reported in a more recent meta-analysis by Holm et al., who included 50 RCTs performed from January 1966 to December 2029 enrolling a total of 19,004 patients with microbiologically-documented SAF (Holm et al., 2020). In most of the RCTs, short-term (≤5 days) administration of cephalosporins and macrolides was compared with the longer course (≥7 days) of penicillin V. In some other trials comparison was made between different duration of penicillin V administration (5 days vs. 10 days) or between the short-term course of various broad-spectrum antibiotics such as cephalosporins and macrolides. Overall evaluation of the studies showed that short-term antibiotic therapy was as effective as long-term therapy in inducing early clinical cure (OR, 0.95; 95% CI, 0.79–1.15). Moreover, early bacteriological eradication was more common in children receiving short-term therapy, although difference was not statistically significant (OR, 0.78; 95% CI, 0.60–1.00). However, important differences emerged when antibiotic groups were directly compared: It was observed that penicillin V was more effective when given for a longer period for both early clinical cure (OR, 0.43; 95% CI, 0.23–0.82) and early microbiological eradication (OR, 0.34; 95% CI, 0.19–0.61), that short-course cephalosporins were more effective than long-term penicillin V administration for both the primary outcomes (OR 1.48, 95% CI 1.11–1.96 for early clinical cure and OR 1.60, 95% CI 1.10–1.79 for early microbiological eradication) and that short-course macrolides were as effective as the long-term penicillin V course (OR 0.76; 95% CI, 0.48–1.20). No difference between groups was observed on late clinical cure (OR 0.91; 95% CI, 0.80–1.04). On the contrary, when adverse events were calculated, it was evidenced that children receiving short-term therapy were at higher risk (17.7% vs. 12.3%) except for children treated with penicillin V that experienced more adverse events when given long-term therapy (33% vs. 23%). As in the case of the previously cited meta-analysis (Altamimi et al., 2009), results of the study by Holm et al. should be considered with extreme caution (Holm et al., 2020). As reported by the authors themselves, most of the included studies had high risk of bias mainly because lack of blinding of patients, personnel, and evaluators. Moreover, the study itself has important limitations that do not allow to draw firm conclusions. Only studies carried out in developed countries were included in the meta-analysis. This makes it difficult to generalize results, especially for the impact of short-term therapy on the incidence of ARF that has very different incidence in developing countries compared to the industrialized world. Moreover, the meta-analysis did not consider the role of different antibiotic dosages in conditioning clinical and microbiological findings. For example, when short-term and long-term penicillin V regimens were compared, all the patients receiving this antibiotic were grouped according to treatment duration without any consideration of daily drug dosage and fractioning. As penicillin V regimens varied significantly, it cannot be excluded that in some cases short-term therapy was more or equally effective than the usual long-term therapy, but this effect was masked by the negative studies. Penicillin V is a time-dependent antibiotic, and it can be assumed that administration schedules assuring for few days serum antibiotic levels persistently above the *S. pyogenes* minimum inhibitory concentration could be more effective than the recommended 10-day course, although the drug was given for a shorter period (Reed, 2000). Consistent with this hypothesis is the evidence that 800 mg penicillin V four times daily for 5 days was non-inferior in clinical outcome to 1 g penicillin V three times daily for 10 days in adults (Skoog Ståhlgren et al., 2019). Time to relief of symptoms was shorter in the 5-day group, early clinical cure was similar (89.6% vs. 93.3%) as it was early bacteriological eradication (80.4% vs. 90.7%).

Further data regarding the optimal duration of antibiotic treatment for ASF were recently reported in a Cochrane review examining paper published until September 2020 and enrolling patient of any age, mostly children, with ASF (Reed, 2000). Main aim of the study was to compare short-term therapy with cephalosporins or macrolides to traditional 10-day therapy with penicillin V in alleviating symptoms, shortening the illness duration, preventing clinical relapse and acute and late complications. A total of 19 double-blind RCTs reported in 18 publications with 5,839 participants were analyzed. Results of the study were disappointing as they did not solve any of the open problems regarding ASF treatment. Included studies were very heterogeneous and, generally, of poor quality, mainly due to the lack or poor reporting of randomization, allocation concealment, and blinding. Impact on late complications could not be evaluated due to the very reduced number of complicated cases in all the studied groups. Effect on acute clinical manifestations could not be precisely established because of the heterogenicity of the studies, the poor report of clinical manifestations and the different methods of collection of signs and symptoms during therapy and after its end. The authors declared that they were unable to decide whether there were clinically relevant differences in symptoms resolution between short-term treatment with cephalosporins or macrolides and traditional 10-day penicillin V treatment. Only studies evaluating loracarbef suggested that short-term courses of this drug could more effective than penicillin V for symptoms resolution in children (OR, 0.70, 95% CI, 0.49–0.99) (van Driel et al., 2021).

Starting from these data, it could be concluded that available studies do not definitively support the use of short-term therapy for ASF. Anyway, even if one or more cephalosporins or macrolides given for a shorter period were definitively found as effective as to the traditional penicillin V 10-day course, the increased risk of drug-related adverse events and the increased economic cost of therapy should make physicians seriously consider the opportunity to use these newer antibiotics. As pointed out by World Health Organization (World Health Organization, 2023b), macrolides and cephalosporins are broad spectrum drugs and should be prescribed only when the first-line drugs, as in this case penicillin V or amoxicillin, fail. To improve knowledge on short-term therapy of ASF, short-term administration of penicillin V or amoxicillin, eventually with increased daily dosage or with different total daily dose fractioning, should be further studied.

## 3 Acute otitis media (AOM)

The large use of pneumococcal conjugate vaccines (Principi and Esposito, 2021; Hu et al., 2022) and influenza vaccines (Principi et al., 2012; Norhayati et al., 2017) has significantly reduced AOM incidence. However, AOM remains a very common disease in pediatrics. Most children experience at least one episode of AOM in the first 3 years of life and about one-third suffer from recurrent episodes (Marchisio et al., 2017; Venekamp et al., 2017). As the majority of the cases are due to bacteria, AOM is the most common pediatric condition for which antibiotics are prescribed (McCaig et al., 2002; Grijalva et al., 2009). On the other hand, several studies have reported that children with AOM benefit from antimicrobial treatment as compared with placebo (Hoberman et al., 2011; Tähtinen et al., 2011; Principi and Esposito, 2020). The duration of pain and the risk of treatment failure are significantly decreased Treatment failure is reduced by 62% (Hoberman et al., 2011; Tähtinen et al., 2011; Principi and Esposito, 2020) and, at 2–7 days after presentation, the risk of pain is reduced by about 40% (Del Mar et al., 1997).

Fortunately, several AOM cases, particularly those with mild to moderate severity occurring in children older than 2 years of age, solve spontaneously. This has led several scientific societies in Europe (Marchisio et al., 2019; Suzuki et al., 2020) America (Lieberthal et al., 2013; Le Saux et al., 2016) and Asia (Kitamura et al., 2015) to recommend that children ≥ 2 years old with mild to moderately bulging tympanic membrane and mild otalgia who have a low-grade fever and do not appear severely ill are managed with a watchful waiting period of 24–48 h if timely reassessment is possible and antibiotic can be prescribed if child worsen or fail to improve. Treatment should be reserved to children with severe or complicated AOM of any age and in children <2 years with bilateral AOM regardless of disease severity (Lieberthal et al., 2013; Kitamura et al., 2015; Le Saux et al., 2016; Marchisio et al., 2019; Suzuki et al., 2020).

Suggestions for duration of antibiotic administration vary according with the age of the patients and characteristics of AOM. For severe, complicated and recurrent cases, a 10-day course is generally recommended. Debated remains, on the contrary, the most effective duration of antibiotic administration in children with uncomplicated AOM. For patients aged ≥ 2 years for whom watchful waiting is not possible, many experts consider appropriate a 5 to 7 days-course of antibiotic therapy. This because in these subjects a short course was found not substantially different from a 10 days-course in avoiding persistence of symptoms, relapse or recurrence (Cohen et al., 2001; Pichichero et al., 2001). Several factors could explain the comparability between a short and long course of antibiotics. Among them: 1) the enrollment among the studied patients of children without AOM but with diseases not requiring antibiotic therapy, given the difficulty of diagnosing AOM in outpatient practice; 2) the spontaneous cure of cases of AOM although of bacterial origin; 3) the eradication of bacteria from the middle ear after a few days of antibiotic treatment; and 4) the poor penetration of antibiotics into the middle ear after a few days of treatment when the inflammation is reduced (Cohen et al., 2001). However, results of a meta-analysis of 49 trials including 12,045 participants could lead to different conclusions and suggest a preference for long-term therapy. In this study administration of antibiotics for 5 days was associated with a slight but significant ly increased risk of failure (21% vs. 18%; OR 1.34, 95% CI 1.15–1.55) at 1 month initiation of therapy (Kozyrskyj et al., 2010).

Not perfectly defined is also the best duration of therapy for children <2 years old. In a study enrolling 520 children, 6–23 months of age, with AOM randomized to receive amoxicillin-clavulanic acid for 10 or 5 days (Hoberman et al., 2016), clinical failure was significantly more common in patients treated with short-term therapy (34% vs. 16%) than in those receiving treatment for 10 days. Difference was even greater when only children with probably severe illness were considered. Moreover, the percentage of children whose symptom scores decreased more than 50% from the beginning to the end of antibiotic administration was lower in the 5-day group than in the 10-day group (80% vs. 91%, *p* = 0.003) (Hoberman et al., 2016). Rates of recurrences, adverse events, or nasopharyngeal colonization with penicillin-nonsusceptible bacteria were not different between groups. Despite this, the authors concluded suggesting long-term administration of antibiotics as the best solution for AOM treatment in younger children (Hoberman et al., 2016). However, despite well-designed and rigorously conducted, this study leaves open many questions about the best therapy for AOM of children <2 years old. In this study, amoxicillin-clavulanic acid was used. This is not the recommended therapy for uncomplicated AOM. Amoxicillin is generally recommended as first line drug worldwide. Studies with amoxicillin are needed to definitively decide whether AOM of younger children require specific antibiotic choices. Moreover, in the study, despite in a lower proportion than among those receiving long-term therapy, a not marginal number of children given short-term therapy was cured (Hoberman et al., 2016). On the other hand, epidemiological studies have shown that spontaneous resolution of AOM can occur in a relevant number of children <2 years of age. This means that even short-term therapy can be effective and would be important to identify which children <2 years do not need long-term therapy. If the reasons for reducing the duration of antibiotic therapy is to limit antibiotic abuse and related problems, including development of antimicrobial resistance and increased risk of future infections (Rovers et al., 2006), it is mandatory to prescribe drugs with the narrowest spectrum of antibacterial activity and identify the patients that can be cured by the shortest period of drug administration (Venekamp and Schilder, 2017). Further studies in this regard are needed.

## 4 Community-acquired pneumonia (CAP)

CAP is a very common cause of pediatric morbidity and hospitalization worldwide (Roh et al., 2022). Although frequency and severity of CAP are several times higher in the developing world, they remain significant in all the industrialized countries. For example, in the northern Europe, incidence of CAP was recently found to be 32.8–33.8 cases per 10,000 children <5 years old or 14.4–14.7 cases per 10,000 children <16 years old (Senstad et al., 2009). Recommendations for duration of antibiotic therapy in children with CAP differ among institutions and scientific societies. The World Health Organization suggests 3 days for CAP with fast breathing and 5 days in case of chest indrawing (World Health Organization, 2023c). On the contrary, several national guidelines, mainly those prepared in industrialized countries, including those by the Pediatric Infectious Disease Society of the United States (Bradley et al., 2011), the British Thoracic Society (Harris et al., 2011) and the European Society for Paediatric Infectious Diseases (Esposito et al., 2012) recommend a longer treatment, generally of 7–10 days. Use of different criteria to diagnose CAP seems to be the main reason for this difference. In WHO studies only clinical criteria were used. Unfortunately, accuracy of symptoms and physical examination findings in identifying children with CAP is poor (Shah et al., 2017). Several common lower respiratory tract infections (LRTIs) of viral origin, mainly bronchiolitis and infectious wheezing, mimic CAP clinical manifestations. The enrollment of several cases of patients not requiring antibiotics among those with presumed CAP may have greatly influenced final results of WHO studies (World Health Organization, 2023c), leading to the conclusion that a very short-term antibiotic therapy could be sufficient to cure all CAPs, including those due to bacteria. On the contrary, in industrialized countries, some efforts for a more accurate identification of CAP cases and, among these, of those of bacterial origin have been made. Laboratory tests, chest radiograph and respiratory secretion culture were included among the criteria used to diagnose CAP (Esposito et al., 2022). For example, in a study carried out in Israel only cases with radiological evidence of alveolar CAP were enrolled (Greenberg et al., 2014). Although lung consolidation has been described even in viral cases, it is more common in bacterial CAP and is considered a reliable marker for the identification of pneumococcal CAP (Berce et al., 2019). The inclusion of a greater number of cases requiring antibiotics among those enrolled in the studies may have influenced results of treatment, justifying suggestions for a longer antibiotic administration. On the other hand, in the Israeli study a 3-day course with high-dose oral amoxicillin was associated with a 40% failure rate, whereas no case of treatment failure was evidenced in children given a 10 days-course (Greenberg et al., 2014).

In order to definitively solve the problem of the optimal duration of antibiotic administration for CAP therapy in children, in recent years several studies were carried out. In complicated CAP cases requiring hospitalization it was generally state that duration of antibiotic administration should have been individualized mainly on the basis of patient and disease characteristics. Ten days or even longer period of therapy for very complicated cases were suggested (Esposito et al., 2022). Different results were obtained when children with mild to moderate CAP followed in the outpatient setting were studied. In most of these trials in the intent to compare efficacy of short- and long-term therapy not only failure rates, already considered the primary outcome in all the previous studies, but also other variables depending on antibiotic use were evaluated. Incidence of drug-related adverse events, including diarrhea, emergence of bacterial resistance, and impact of CAP on caregivers and child quality of life were studied. Generally, no difference in efficacy between short-term and long-term studies was evidenced Moreover, in some studies short-term antibiotic administration was associated with a lower incidence of drug-related adverse events, with some microbiological advantages and with lower impact on patient and family life (Marques et al., 2022; Kuitunen et al., 2023). Most of these findings are clearly evidenced in the well conducted systematic review and meta-analysis by Li et al. (Li et al., 2022), . Data regarding a total of 9 RCTs published until 3 March 2022, that included 11,143 otherwise healthy children were evaluated. Most of the patients (98%) aged less than 5 years. Diagnosis of CAP was based only on clinical manifestations in 6 studies. Chest Xray findings were taken into account in two studies. Finally, in one of the studies considering cotrimoxazole, criteria for CAP diagnosis were not detailed. Amoxicillin (7 studies) or cotrimoxazole (2 studies) were tested. Standard (35–50 mg/kg/day) and high dose (75–100 mh/kg/day) of amoxicillin given for 3, 5 and 10 days in 2 or 3 daily administrations were compared. When reported, cotrimoxazole dosage was 40 mg and 80 mg twice daily in children 2–12 months and ≥12 months, respectively, for a total of 3 or 5 days. Treatment failure, defined as persistence or worsening of clinical manifestations at the end of therapy, occurred in 12.8% vs. 12.6% of participants randomized to a shorter vs. a longer course of antibiotics, respectively. A 3-day course was noninferior to a 5-day course (RR, 1.01; 95% CI, 0.91–1.12), and a 5-day course was noninferior to a 10-day course (RR, 0.87; 95% CI, 0.50–1.53). When risk of relapse, defined as recurrence of any sign of CAP among patient who had been clinically cured, was calculated, it was shown that a shorter course of oral antibiotics was noninferior to a longer course [relative risk (RR), 1.12; 95% CI, 0.94–1.34]. However, subgroup analysis revealed that noninferiority of short-term therapy to long-term therapy for risk of failure or recurrence was met for children aged less than 5 years (RR, 1.01; 95% CI, 0.91–1.11) but not for older children (RR, 2.07; 95% CI, 0.76–5.63). Drug-related gastrointestinal adverse events and caregiver absenteeism were less common in children given a shorter course of antibiotics (RR, 0.79; 95% CI, 0.66–0.95, and RR 0.74; 95% CI, 0.65–0.84, respectively). Finally, in one of the studies it was shown that the short-course therapy was associated with a lower risk of development of bacterial resistance to the administered drug among pharyngeal bacteria (Williams et al., 2022). After therapy, antibiotic resistance genes in prokaryotic cells from throat swab were significantly lower in children treated with short-course therapy than in those who received long-term therapy. The quantity of antibiotic resistant genes, expressed as resistance genes per prokaryotic cell, was 1.17 (range of resistance genes per cell, 0.35–2.43) and 1.33 (range of resistance genes per cell, 0.46–11.08, *p* = 0.01), respectively. Similar microbiological findings were also reported in the study by Pettingrew et al. (Pettigrew et al., 2022). All these findings seem to suggest that children with mild to moderate CAP can be treated with a short course of antibiotics, mainly amoxicillin for 5 days, without significant risk of treatment failure or recurrence and with several advantages in term of reduction of antibiotic-related adverse events and costs and emergence of resistant respiratory bacteria. As a consequence, some experts have suggested that scientific societies that still recommend longer therapy should revise pediatric CAP guidelines, recommending short-term therapy for all the children with mild to moderate disease (BMJ Publishing Group Ltd and Royal College of Paediatrics and Child Health, 2023). However despite favoring a significant reduction of antibiotic consumption in a significant number of children with CAP, this recommendation may be criticized. Reduction is suggested for all the children, without any selection according to the severity of disease. In all the studies mild and moderate CAP cases are pooled and subgroup analysis was never made. Similarly, no attention was paid to CAP etiology. Differences related to the type of prescribed antibiotic and schedule of administration were rarely evaluated (Bielicki et al., 2021). Finally, currently available data do not allow recommending a reduction in the duration of antibiotic therapy in children aged >5 years. The results of the LI meta-analysis (Li et al., 2022) are negative in this regard and, in any case, collected on too small a number of subjects to be considered definitive. Furthermore, the evidence that most of children <5 years can be treated for 5 days without clinical problems may derive from the fact that the vast majority of them suffer from a viral disease that does not need antibiotic therapy. This may suggest a different approach for these CAPs, an approach that, if proven effective, could reduce even more the consumption of antibiotics. In these cases what has already been suggested for the treatment of AOM could be applied, i.e., watchful waiting. The child could simply be followed for the first 48–72 h and treated only if the clinical conditions did not improve during or at the end of this period. In this way, many children would not be treated, and the consumption of antibiotics drastically reduced. Obviously, this presupposes the consent of the parents and the careful and continuous monitoring of the patient’s conditions for the whole time of watchful waiting. In the studies enrolling children diagnosed and treated in the outpatient setting, all To decide the optimal duration of therapy in children with mild to moderate CAP, further studies able to characterize efficacy of short-term therapy in the different subgroups of children with uncomplicated CAP are urgently needed.

## 5 Conclusion

Reducing the duration of antibiotic therapy for some of the most common pediatric respiratory infections could be a valid measure to contain the antibiotic abuse and related problems, including the emergence of AMR. Several data seem to indicate that this type of intervention is possible, as short-term therapy has been found as effective as the traditionally recommended long-term therapy in some studies regarding ASF, AOM and mild to moderate CAP. However, for none of these respiratory infections, available data allow to definitively decide which children with these diseases can be treated with a short-term therapy, which antibiotics and which schedule of administration assure the best results, avoiding risk of failure, relapse or recurrence. Further studies are needed to solve these problems.

For ASF, several attempts to substitute the traditional prescription for 10 days of penicillin V or amoxicillin have been made. A large number of antibiotics potentially effective against *Sp* given with different administration schedules were compared with each other or with penicillin V or amoxicillin. Moreover, short- and long-term administration of penicillin V and amoxicillin were studied. Unfortunately, no definitive conclusion about the best solution for short-term therapy is presently available. Until it is clearly shown how to safely reduce the duration of the antibiotic therapy for ASF traditional recommendations suggestion 10 days-course of penicillin V or amoxicillin should be followed. Different choices are at risk of inducing more harm than good. The significant increase in drug-related adverse events and economic costs of therapy shown in several studies in which cephalosporins and macrolides were tested indicates that attention should be paid in this regard.

For OMA cases, experts agree that short-term therapy can be used for older children with at low risk of negative evolution, despite some negative results and the lack of data regarding the best drug and the best schedule. However, these cases are those that usually solve spontaneously. The widest possible use of the watchful waiting strategy seems the best solution to limit antibiotic use in these subjects. Finally, some doubts remain on the best therapy for younger children as it is not defined whether all these patients need long-term therapy and what is the most appropriate drug. In these cases, a 10 days-course of amoxicillin remains the best solution to assure disease cure.

Regarding CAP, the option to reduce duration of antibiotic administration in children with mild to moderate disease aged ≤5 years seems clearly demonstrated by several studies and should be adopted in clinical practice. A better definition of the role of severity in conditioning response to short-term therapy as well as of the possibility to use point-of-care biomarkers could better define patients who can have short-term treatment without any significant risk of negative evolution. However, this practice cannot be used in older children as no reliable data on the true efficacy of short-term therapy in these subjects have been collected. Moreover, considering that most of the mild to moderate CAP cases developing in younger children are due to viruses and can solve spontaneously, an even more innovative approach to the mild to moderate CAPs of these subjects could be proposed and tested. The watchful waiting strategy already suggested for AOM could be used. If effective an even greater reduction of antibiotic consumption could be obtained.

In conclusion, further researches are needed to better establish characteristics of pediatric patients who can have benefit with short-term therapy in common bacterial respiratory infections. These studies are urgently required because any initiative to reduce abuse and misuse of antibiotics should be strongly supported in order to limit all the AMR-related problems.

## References

[B1] Akram F. Imtiaz M. Haq I. U. (2023). Emergent crisis of antibiotic resistance: A silent pandemic threat to 21st century. Microb. Pathog. 174, 105923. 10.1016/j.micpath.2022.105923 36526035

[B2] Aljeldah M. M. (2022). Antimicrobial resistance and its spread is a global threat. Antibiot. (Basel) 11, 1082. 10.3390/antibiotics11081082 PMC9405321 36009948

[B3] Altamimi S. Khalil A. Khalaiwi K. A. Milner R. Pusic M. V. Al Othman M. A. (2009). Short versus standard duration antibiotic therapy for acute streptococcal pharyngitis in children. Cochrane Database Syst. Rev. 1, CD004872. 10.1002/14651858.CD004872.pub2 19160243

[B4] Antimicrobial Resistance Collaborators (2022). Global burden of bacterial antimicrobial resistance in 2019: A systematic analysis. Lancet 399, 629–655. 10.1016/S0140-6736(21)02724-0 35065702 PMC8841637

[B5] ASPE. National (2023). Action plan for combating antibiotic-resistant bacteria, 2020-2025. Available at: https://aspe.hhs.gov/reports/national-action-plan-combating-antibiotic-resistant-bacteria-2020-2025 (Accessed February 10, 2023).

[B6] Berce V. Tomazin M. Gorenjak M. Berce T. Lovrenčič B. (2019). The usefulness of lung ultrasound for the aetiological diagnosis of community-acquired pneumonia in children. Sci. Rep. 9, 17957. 10.1038/s41598-019-54499-y 31784642 PMC6884636

[B7] Bielicki J. A. Stöhr W. Barratt S. Dunn D. Naufal N. Roland D. (2021). Effect of amoxicillin dose and treatment duration on the need for antibiotic Re-treatment in children with community-acquired pneumonia: The CAP-IT randomized clinical trial. JAMA 326, 1713–1724. 10.1001/jama.2021.17843 34726708 PMC8564579

[B8] BMJ Publishing Group Ltd and Royal College of Paediatrics and Child Health (2023). Short courses of antibiotics for community acquired pneumonia. Arch. Dis. Childh 108, 52. 10.1136/archdischild-2022-325186 36521859

[B9] Bradley J. S. Byington C. L. Shah S. S. Alverson B. Carter E. R. Harrison C. (2011). Executive summary: The management of community-acquired pneumonia in infants and children older than 3 months of age: Clinical practice guidelines by the pediatric infectious diseases society and the infectious diseases society of America. Clin. Infect. Dis. 53, 617–630. 10.1093/cid/cir625 21890766 PMC3202323

[B10] Cassini A. Högberg L. D. Plachouras D. Quattrocchi A. Hoxha A. Simonsen G. S. (2019). Attributable deaths and disability-adjusted life-years caused by infections with antibiotic-resistant bacteria in the EU and the European economic area in 2015: A population-level modelling analysis. Lancet Infect. Dis. 19, 56–66. 10.1016/S1473-3099(18)30605-4 30409683 PMC6300481

[B11] Centers for Disease Control and Prevention (2023). Group A streptococcal (GAS) disease. Pharyngitis (strep throat). Available at: https://www.cdc.gov/groupastrep/diseases-hcp/strep-throat.html (Accessed February 6, 2023).

[B12] Chiappini E. Principi N. Mansi N. Serra A. De Masi S. Camaioni A. (2012). Management of acute pharyngitis in children: Summary of the Italian national institute of health guidelines. Clin. Ther. 34, 1442–1458.e2. 10.1016/j.clinthera.2012.04.028 22691611

[B13] Chiappini E. Regoli M. Bonsignori F. Sollai S. Parretti A. Galli L. (2011). Analysis of different recommendations from international guidelines for the management of acute pharyngitis in adults and children. Clin. Ther. 33, 48–58. 10.1016/j.clinthera.2011.02.001 21397773

[B14] Cohen R. Ovetchkine P. Géhanno P. (2001). Current approaches to otitis media. Curr. Opin. Infect. Dis. 14, 337–342. 10.1097/00001432-200106000-00015 11964853

[B15] Costelloe C. Metcalfe C. Lovering A. Mant D. Hay A. D. (2010). Effect of antibiotic prescribing in primary care on antimicrobial resistance in individual patients: Systematic review and meta-analysis. BMJ 340, c2096. 10.1136/bmj.c2096 20483949

[B16] Curran J. Lo J. Leung V. Brown K. Schwartz K. L. Daneman N. (2022). Estimating daily antibiotic harms: An umbrella review with individual study meta-analysis. Clin. Microbiol. Infect. 28, 479–490. 10.1016/j.cmi.2021.10.022 34775072

[B17] Davar K. Clark D. Centor R. M. Dominguez F. Ghanem B. Lee R. (2022). Can the future of ID escape the inertial dogma of its past? The exemplars of shorter is better and oral is the new IV. Open Forum Infect. Dis. 10, ofac706. 10.1093/ofid/ofac706 36694838 PMC9853939

[B18] De Luca M. Donà D. Montagnani C. Lo Vecchio A. Romanengo M. Tagliabue C. (2016). Antibiotic prescriptions and prophylaxis in Italian children. Is it time to change? Data from the ARPEC project. PLoS One 11, e0154662. 10.1371/journal.pone.0154662 27182926 PMC4868290

[B19] Del Mar C. Glasziou P. Hayem M. (1997). Are antibiotics indicated as initial treatment for children with acute otitis media? A meta-analysis. BMJ 314, 1526–1529. 10.1136/bmj.314.7093.1526 9183201 PMC2126788

[B20] Del Mar C. B. Glasziou P. P. Spinks A. B. (2006). Antibiotics for sore throat. Cochrane Database Syst. Rev. 4, CD000023. 10.1002/14651858.CD000023 11034668

[B21] English B. K. Gaur A. H. (2010). The use and abuse of antibiotics and the development of antibiotic resistance. Adv. Exp. Med. Biol. 659, 73–82. 10.1007/978-1-4419-0981-7_6 20204756

[B22] Esposito S. Cohen R. Diez Domingo J. Falup Pecurariu O. Greenberg D. Heininger U. (2012). Antibiotic therapy for pediatric community-acquired pneumonia: Do we know when, what and for how long to treat? Pediatr. Infect. Dis. J. 31, e78–e85. 10.1097/INF.0b013e318255dc5b 22466326

[B23] Esposito S. Dal Canto G. Caramia M. R. Fainardi V. Pisi G. Principi N. (2022). Complications in community acquired pneumonia: Magnitude of problem, risk factors, and management in pediatric age. Expert Rev. Anti Infect. Ther. 20, 45–51. 10.1080/14787210.2021.1927710 33971782

[B24] Esposito S. Tagliabue C. Picciolli I. Semino M. Sabatini C. Consolo S. (2011). Procalcitonin measurements for guiding antibiotic treatment in pediatric pneumonia. Respir. Med. 105, 1939–1945. 10.1016/j.rmed.2011.09.003 21959024

[B25] European Commission (2023). A European one health action plan against antimicrobial resistance (AMR). Available at: https://health.ec.europa.eu/system/files/2020-01/amr_2017_action-plan_0.pdf (Accessed February 10, 2023).

[B26] Fanelli U. Pappalardo M. Chinè V. Gismondi P. Neglia C. Argentiero A. (2020). Role of artificial intelligence in fighting antimicrobial resistance in pediatrics. Antibiot. (Basel) 9, 767. 10.3390/antibiotics9110767 PMC7692722 33139605

[B27] GBD 2019 Diseases and Injuries Collaborators (2020). Global burden of 369 diseases and injuries in 204 countries and territories, 1990-2019: A systematic analysis for the global burden of disease study 2019. Lancet 396, 1204–1222. 10.1016/S0140-6736(20)30925-9 33069326 PMC7567026

[B28] Greenberg D. Givon-Lavi N. Sadaka Y. Ben-Shimol S. Bar-Ziv J. Dagan R. (2014). Short-course antibiotic treatment for community-acquired alveolar pneumonia in ambulatory children: A double-blind, randomized, placebo-controlled trial. Pediatr. Infect. Dis. J. 33, 136–142. 10.1097/INF.0000000000000023 23989106

[B29] Grijalva C. G. Nuorti J. P. Griffin M. R. (2009). Antibiotic prescription rates for acute respiratory tract infections in US ambulatory settings. JAMA 302, 758–766. 10.1001/jama.2009.1163 19690308 PMC4818952

[B30] Harris M. Clark J. Coote N. Fletcher P. Harnden A. McKean M. (2011). British thoracic society guidelines for the management of community acquired pneumonia in children: Update 2011. Thorax 66, ii1–23. 10.1136/thoraxjnl-2011-200598 21903691

[B31] Hoberman A. Paradise J. L. Rockette H. E. Kearney D. H. Bhatnagar S. Shope T. R. (2016). Shortened antimicrobial treatment for acute otitis media in young children. N. Engl. J. Med. 375, 2446–2456. 10.1056/NEJMoa1606043 28002709 PMC5319589

[B32] Hoberman A. Paradise J. L. Rockette H. E. Shaikh N. Wald E. R. Kearney D. H. (2011). Treatment of acute otitis media in children under 2 years of age. N. Engl. J. Med. 364, 105–115. 10.1056/NEJMoa0912254 21226576 PMC3042231

[B33] Holm A. E. Llor C. Bjerrum L. Cordoba G. (2020). Short-vs. Long-course antibiotic treatment for acute streptococcal pharyngitis: Systematic review and meta-analysis of randomized controlled trials. Antibiot. (Basel) 9, 733. 10.3390/antibiotics9110733 PMC7692631 33114471

[B34] Hu T. Done N. Petigara T. Mohanty S. Song Y. Liu Q. (2022). Incidence of acute otitis media in children in the United States before and after the introduction of 7- and 13-valent pneumococcal conjugate vaccines during 1998-2018. BMC Infect. Dis. 22, 294. 10.1186/s12879-022-07275-9 35346092 PMC8962537

[B35] Kitamura K. Iino Y. Kamide Y. Kudo F. Nakayama T. Suzuki K. (2015). Clinical practice guidelines for the diagnosis and management of acute otitis media (AOM) in children in Japan - 2013 update. Auris Nasus Larynx 42, 99–106. 10.1016/j.anl.2014.09.006 25450605

[B36] Kozyrskyj A. Klassen T. P. Moffatt M. Harvey K. (2010). Short-course antibiotics for acute otitis media. Cochrane Database Syst. Rev. 9, CD001095. 10.1002/14651858.CD001095.pub2 PMC7052812 20824827

[B37] Kuitunen I. Jääskeläinen J. Korppi M. Renko M. (2023). Antibiotic treatment duration for community-acquired pneumonia in outpatient children in high-income countries-A systematic review and meta-analysis. Clin. Infect. Dis. 76, e1123–e1128. 10.1093/cid/ciac374 35579504 PMC9907524

[B38] Le Saux N. Robinson J. L. Canadian Paediatric Society, Infectious Diseases and Immunization Committee (2016). Management of acute otitis media in children six months of age and older. Paediatr. Child. Health 21, 39–50. 10.1093/pch/21.1.39 26941560 PMC4758427

[B39] Lee R. A. Stripling J. T. Spellberg B. Centor R. M. (2023). Short-course antibiotics for common infections: What do we know and where do we go from here? Clin. Microbiol. Infect. 29, 150–159. 10.1016/j.cmi.2022.08.024 36075498

[B40] Li Q. Zhou Q. Florez I. D. Mathew J. L. Shang L. Zhang G. (2022). Short-course vs long-course antibiotic therapy for children with nonsevere community-acquired pneumonia: A systematic review and meta-analysis. JAMA Pediatr. 176, 1199–1207. 10.1001/jamapediatrics.2022.4123 36374480 PMC9664370

[B41] Lieberthal A. S. Carroll A. E. Chonmaitree T. Ganiats T. G. Hoberman A. Jackson M. A. (2013). The diagnosis and management of acute otitis media. Pediatrics 131, e964–e999. 10.1542/peds.2012-3488 23439909

[B42] Marchisio P. Bortone B. Ciarcià M. Motisi M. A. Torretta S. Castelli Gattinara G. (2019). Updated guidelines for the management of acute otitis media in children by the Italian society of pediatrics: Prevention. Pediatr. Infect. Dis. J. 38 (12), S22–S36. 10.1097/INF.0000000000002430 31876602

[B43] Marchisio P. Esposito S. Picca M. Baggi E. Terranova L. Orenti A. (2017). Prospective evaluation of the aetiology of acute otitis media with spontaneous tympanic membrane perforation. Clin. Microbiol. Infect. 23, 486.e1–486.e6. 10.1016/j.cmi.2017.01.010 28110050

[B44] Marques R. I. Calvi P. I. Cruz A. S. Sanchez M. F. L. Baroni F. I. Oommen C. (2022). Shorter versus longer duration of amoxicillin-based treatment for pediatric patients with community-acquired pneumonia: A systematic review and meta-analysis. Eur. J. Pediatr. 181, 3795–3804. 10.1007/s00431-022-04603-8 36066660

[B45] McCaig L. F. Besser R. E. Hughes J. M. (2002). Trends in antimicrobial prescribing rates for children and adolescents. JAMA 287, 3096–3102. 10.1001/jama.287.23.3096 12069672

[B46] Miller K. M. Carapetis J. R. Van Beneden C. A. Cadarette D. Daw J. N. Moore H. C. (2022). The global burden of sore throat and group A Streptococcus pharyngitis: A systematic review and meta-analysis. EClinicalMedicine 48, 101458. 10.1016/j.eclinm.2022.101458 35706486 PMC9124702

[B47] Norhayati M. N. Ho J. J. Azman M. Y. (2017). Influenza vaccines for preventing acute otitis media in infants and children. Cochrane Database Syst. Rev. 10, CD010089. 10.1002/14651858.CD010089.pub3 29039160 PMC6485791

[B48] Papan C. Argentiero A. Porwoll M. Hakim U. Farinelli E. Testa I. (2022). A host signature based on TRAIL, IP-10, and CRP for reducing antibiotic overuse in children by differentiating bacterial from viral infections: A prospective, multicentre cohort study. Clin. Microbiol. Infect. 28, 723–730. 10.1016/j.cmi.2021.10.019 34768022

[B49] Pelucchi C. Grigoryan L. Galeone C. Esposito S. Huovinen P. Little P. (2012). Guideline for the management of acute sore throat. Clin. Microbiol. Infect. 18 (1), 1–28. 10.1111/j.1469-0691.2012.03766.x 22432746

[B50] Pettigrew M. M. Kwon J. Gent J. F. Kong Y. Wade M. Williams D. J. (2022). Comparison of the respiratory resistomes and microbiota in children receiving short versus standard course treatment for community-acquired pneumonia. mBio 13, e0019522. 10.1128/mbio.00195-22 35323040 PMC9040816

[B51] Pichichero M. E. Marsocci S. M. Murphy M. L. Hoeger W. Francis A. B. Green J. L. (2001). A prospective observational study of 5-7-and 10-day antibiotic treatment for acute otitis media. Otolaryngol. Head. Neck Surg. 124, 381–387. 10.1067/mhn.2001.114311 11283494

[B52] Principi N. Baggi E. Esposito S. (2012). Prevention of acute otitis media using currently available vaccines. Future Microbiol. 7, 457–465. 10.2217/fmb.12.23 22439723

[B53] Principi N. Esposito S. (2016). Antimicrobial stewardship in paediatrics. BMC Infect. Dis. 16, 424. 10.1186/s12879-016-1772-z 27538503 PMC4989524

[B54] Principi N. Esposito S. (2021). Pneumococcal disease prevention: Are we on the right track? Vaccines (Basel) 9, 305. 10.3390/vaccines9040305 33804822 PMC8063798

[B55] Principi N. Esposito S. (2020). Unsolved problems and new medical approaches to otitis media. Expert Opin. Biol. Ther. 20, 741–749. 10.1080/14712598.2020.1740677 32178551

[B56] Reed M. D. (2000). Optimal antibiotic dosing. The pharmacokinetic-pharmacodynamic interface. Postgrad. Med. 108 (7), 17–24. 10.3810/pgm.12.2000.suppl10.52 19667545

[B57] Rice L. B. (2008). The maxwell Finland lecture: For the duration-rational antibiotic administration in an era of antimicrobial resistance and clostridium difficile. Clin. Infect. Dis. 46, 491–496. 10.1086/526535 18194098

[B58] Roh E. J. Shim J. Y. Chung E. H. (2022). Epidemiology and surveillance implications of community-acquired pneumonia in children. Clin. Exp. Pediatr. 65, 563–573. 10.3345/cep.2022.00374 36265520 PMC9742763

[B59] Romandini A. Pani A. Schenardi P. A. Pattarino G. A. C. De Giacomo C. Scaglione F. (2021). Antibiotic resistance in pediatric infections: Global emerging threats, predicting the near future. Antibiot. (Basel) 10, 393. 10.3390/antibiotics10040393 PMC8067449 33917430

[B60] Rovers M. M. Glasziou P. Appelman C. L. Burke P. McCormick D. P. Damoiseaux R. A. (2006). Antibiotics for acute otitis media: A meta-analysis with individual patient data. Lancet 368, 1429–1435. 10.1016/S0140-6736(06)69606-2 17055944

[B61] Senstad A. C. Surén P. Brauteset L. Eriksson J. R. Høiby E. A. Wathne K. O. (2009). Community-acquired pneumonia (CAP) in children in Oslo, Norway. Acta Paediatr. 98, 332–336. 10.1111/j.1651-2227.2008.01088.x 19006533

[B62] Shah S. N. Bachur R. G. Simel D. L. Neuman M. I. (2017). Does this child have pneumonia? The rational clinical examination systematic review. JAMA 318, 462–471. 10.1001/jama.2017.9039 28763554

[B63] Shulman S. T. Bisno A. L. Clegg H. W. Gerber M. A. Kaplan E. L. Lee G. (2012). Clinical practice guideline for the diagnosis and management of group A streptococcal pharyngitis: 2012 update by the infectious diseases society of America. Clin. Infect. Dis. 55, 1279–1282. 10.1093/cid/cis847 23091044

[B64] Skoog Ståhlgren G. Tyrstrup M. Edlund C. Giske C. G. Mölstad S. Norman C. (2019). Penicillin V four times daily for five days versus three times daily for 10 days in patients with pharyngotonsillitis caused by group A streptococci: Randomised controlled, open label, non-inferiority study. BMJ 367, l5337. 10.1136/bmj.l5337 31585944 PMC6776830

[B65] Smith B. J. Heriot G. Buising K. (2020). Antibiotic treatment of common infections: More evidence to support shorter durations. Curr. Opin. Infect. Dis. 33, 433–440. 10.1097/QCO.0000000000000680 33148985

[B66] Suzuki H. G. Dewez J. E. Nijman R. G. Yeung S. (2020). Clinical practice guidelines for acute otitis media in children: A systematic review and appraisal of European national guidelines. BMJ Open 10, e035343. 10.1136/bmjopen-2019-035343 PMC7228535 32371515

[B67] Tähtinen P. A. Laine M. K. Huovinen P. Jalava J. Ruuskanen O. Ruohola A. (2011). A placebo controlled trial of antimicrobial treatment for acute otitis media. N. Engl. J. Med. 364, 116–126. 10.1056/NEJMoa1007174 21226577

[B68] Teshome B. F. Vouri S. M. Hampton N. Kollef M. H. Micek S. T. (2019). Duration of exposure to antipseudomonal beta-lactam antibiotics in the critically ill and development of new resistance. Pharmacother 39, 261–270. 10.1002/phar.2201 PMC6507412 30506852

[B69] van Driel M. L. De Sutter A. I. Thorning S. Christiaens T. (2021). Different antibiotic treatments for group A streptococcal pharyngitis. Cochrane Database Syst. Rev. 3, CD004406. 10.1002/14651858.CD004406.pub3 33728634 PMC8130996

[B70] Venekamp R. P. Damoiseaux R. A. Schilder A. G. (2017). Acute otitis media in children. Am. Fam. Physician 95, 109–110. 28084706

[B71] Venekamp R. P. Schilder A. G. M. (2017). Clinical failure is more common in young children with acute otitis media who receive a short course of antibiotics compared with standard duration. Evid. Based Med. 22, 100. 10.1136/ebmed-2017-110697 28404603

[B72] Williams D. J. Creech C. B. Walter E. B. Martin J. M. Gerber J. S. Newland J. G. (2022). Short-vs standard-course outpatient antibiotic therapy for community-acquired pneumonia in children: The SCOUT-CAP randomized clinical trial. JAMA Pediatr. 176, 253–261. 10.1001/jamapediatrics.2021.5547 35040920 PMC8767493

[B73] World Health Organization (2023). Antimicrobial resistance. Global action plan on antimicrobial resistance. Available at: https://www.emro.who.int/health-topics/drug-resistance/global-action-plan.html (Accessed February 10, 2023).

[B74] World Health Organization (2023). Critically important antimicrobials for human medicine: 6th revision. Available at: https://www.who.int/publications/i/item/9789241515528 (Accessed February 10, 2023).

[B75] World Health Organization (2023). Revised WHO classification and treatment of childhood pneumonia at health facilities. Available at: https://apps.who.int/iris/bitstream/handle/10665/137319/9789241507813_eng.pdf;jsessionid=79B4F0183FB32C3C57ED7F65D2B5478B?sequence=1 (Accessed May 2, 2023).

[B76] Zay Ya K. Win P. T. N. Bielicki J. Lambiris M. Fink G. (2023). Association between antimicrobial stewardship programs and antibiotic use globally: A systematic review and meta-analysis. JAMA Netw. Open 6, e2253806. 10.1001/jamanetworkopen.2022.53806 36757700 PMC9912134

